# Resetting the Initial Conditions for Calculating Epidemic Spread: COVID-19 Outbreak in Italy

**DOI:** 10.1109/ACCESS.2020.3015923

**Published:** 2020-08-11

**Authors:** Marina Bagić Babac, Vedran Mornar

**Affiliations:** Faculty of Electrical Engineering and ComputingUniversity of Zagreb 37631 10000 Zagreb Croatia

**Keywords:** Epidemic spread, infection rate, mathematical model, SIR, COVID-19, SARS-CoV-2

## Abstract

Confirmed cases of the disease COVID-19 have spread to more than 200 countries and regions of the world within a few months. Although the authorities report the number of new cases on daily basis, there remains a gap between the number of reported cases and actual number of cases in a population. One way to bridge this gap is to gain more in-depth understanding of the disease. In this paper, we have used the recent findings about the clinical courses of inpatients with COVID-19 to reset the initial conditions of the epidemic process in order to estimate more realistic number of cases in the population. By translating the reported cases certain number of days earlier with regard to an average clinical course of the disease, we have obtained much higher number of cases, which suggests that the actual number of infected cases and death rate might have been higher than reported. Based on the outbreak of COVID-19 in Italy, this paper shows an estimate of the number of infected cases based on infection and removal rates from data during the pandemic.

## I. Introduction

Since appearing in Wuhan, China, in December 2019, the novel coronavirus SARS-CoV-2 has spread to more than 200 countries and regions worldwide, according to data compiled by the U.S. based Johns Hopkins University [1]. The outbreak was declared a Public Health Emergency of International Concern on January 30, 2020 and progressed to become a global pandemic on March 11, 2020 [2].

By mid-March 2020, Europe was declared the world’s major epicenter, and Italy was the first country in Europe to witness a widespread outbreak of the SARS-CoV-2 [3]. With over 20,000 deaths during April 2020, Italy has suffered the highest death toll in Europe, and the second highest globally after the United States [4].

Certain health officials believed that the SARS-CoV-2 virus arrived in Italy long before the first case was discovered [5]. Before the first case was reported, there was an unusually high number of pneumonia cases recorded at certain hospital in northern Italy, suggesting it was possible that the patients with the SARS-CoV-2 were treated as if they had a seasonal flu. Health facilities hosting these patients could have become sites for infection, helping proliferate the spread of the virus [6]. Because the virus spread undetected, some officials believed this was the reason for such a high number of cases in the country [7].

The Italian government dealt with the COVID-19 pandemic by issuing a series of decrees that gradually increased restrictions within lockdown areas (so called, *red zones*), which were then expanded until they ultimately applied to the entire country [8]. Since March 9, 2020 Italy’s 60 million residents have been placed on lockdown. With “I stay at home” decree [9], the Italian government declared the entire national territory as protected area where people were requested to move only if necessary (for work or health reasons, or situations of necessity). In addition, people were obliged to wear a mask when leaving their house, and all forms of gathering of people in public places or places open to the public were prohibited throughout the country. Restaurants, bars and most shops were closed since March 12, 2020 with exception of pharmacies and food stores, which had to guarantee distance of one meter between people [10].

In such settings, where much of the disease and its spread remains unknown, it is challenging to predict trends and the end of the epidemic process based only on daily reports of the confirmed, recovered and death cases. However, there are various attempts to model the dynamics of the COVID-19 [11]–[13], but it might still be early to predict what will happen due to the complexity of the biomedical factors and social systems involved [14]. Currently, most of the published work is based on data from mainland China [15]–[17], so our analysis is among the first using the ongoing COVID-19 situation in Italy.

In this paper, we used a traditional infectious disease prediction model based on differential equations (so called, SIR – *Susceptible Infected Removed*) that can reflect the dynamic characteristics of infectious disease according to the laws of transmission within the population [18]. The model assumes that the total number of people in a certain area is a constant, which can prompt the natural transmission process of infectious diseases and reveal the overall information transmission law [19]. In practice, the population is changing over time due to interactions with other populations in terms of food, resources and living space. In addition, it is assumed the connection between individuals is random, and the difference between spreading individuals is ignored, thus limiting the application scope of the model [20].

However, in the light of recent findings about COVID-19 in medicine [21]–[24], it is possible to enrich numerical daily reports on COVID-19 with data relating to the disease. Therefore, we proposed how to incorporate these findings into the mathematical approach to epidemic spread modelling. Our algorithm for resetting the initial conditions of the epidemic process is based on the clinical courses of inpatients with COVID-19. Based on these reset conditions, we estimated the spread of disease using simplified SIR model. During the outbreak, it was essential to monitor the effectiveness of measures taken by governments on the course of the epidemic. In March, there was already a sufficient amount of data collected in Italy to predict certain outcomes of the process. By separating the obtained data into two subsets, one ranging from the beginning of the epidemics, and the other ranging from the government intervention to some days after the epidemics peak, we have shown how the applied intervention reflect on the parameters of the epidemic process.

## II. Research Methodology

### A. Data Collection

The Italian Civil Protection Department had published the cumulative data of confirmed, recovered and death cases on daily basis, which is publicly available online [25]. In addition, WHO (World Health Organization) had published daily Situation Reports [2] with worldwide reported cases on COVID-19. This study made use of these publicly available datasets in order to estimate the real number of infected cases by resetting the initial conditions of epidemic process in Italy.

It is well known that different environmental factors influence the spread of communicable diseases that are prone to cause epidemics. The most important of these are [26]: water supply, sanitation facilities, food and climate. For example, there is some evidence to suggest that pathogens can be spread from one region to another along air streams or by wind. However, this study is limited to only reported cases of the disease in Italy, while predicting the epidemic process given that certain government measures have taken an action.

### B. Resetting the Initial Conditions of Epidemic Process

Recently, Zhou *et al.* [21] have explained the clinical courses of major symptoms and outcomes, and duration of viral shedding from illness onset in patients hospitalized with COVID-19. According to the recent findings [22], the median duration of symptoms and onset of complications and outcomes for a survivor is twenty days, being discharged from hospital on twenty-second day. The median duration of hospital treatment for non-survivors is nineteen days. The patients got admitted to the hospital on the tenth day on average from the illness onset [23].

Therefore, we proposed how to apply the above findings to estimate the trend of an outbreak in Italian population. The first case of COVID-19 in Italy was reported on January 31, 2020, so we translated the date for the confirmed cases by ten days backwards respectively, suggesting that these cases got infected ten days on average before they were reported. The first recovered case in Italy was reported on February 22, 2020 and the first death case was reported on February 24, 2020. Likewise, we translated the recovered cases by twenty-two days backwards, and the death cases by nineteen days backwards. Then, we summed up these translated cases on the respective days. Due to duplication of recovered and death cases (being also reported in time as newly confirmed cases), we removed any given recovered or death case from the translated dataset on the day of their report.

The pseudocode of the explained algorithm in shown in Table 1, where the constants for data shift TIME_C, TIME_R and TIME_D correspond to the current findings about the COVID-19 (10, 22 and 19 days, respectively), but can be updated if the comprehension of the disease changes any time in the future. The *shift(v,t,p)* subroutine in the pseudocode is a vector operation which shifts a vector $v$ exactly $t$ places to the left (position $p =`l'$). The *subtract(a,b)* subroutine subtracts a vector $b$ from vector $a$.

**TABLE 1:** Pseudocode of the COVID-19-SHIFT Algorithm for Resetting the Initial Conditions of an Epidemic Spread

function shift-data (x, y, z, TIME_C, TIME_R, TIME_D)
Input: Three numeric vectors x, y, z
Three constants TIME_C, TIME_C, TIME_D
Output: One numeric vector x’
if (x, y, & z = 0) then return x
else
x’ = shift (x, TIME_C, ‘l’)
y’ = shift (y, TIME_R, ‘l’)
z’ = shift (z, TIME_D, ‘l’)
x’ = sum (x’, y’, z’)
x’ = subtract (x’, y)
x’ = subtract (x’, z)
return x’
end

**FIGURE 1.**
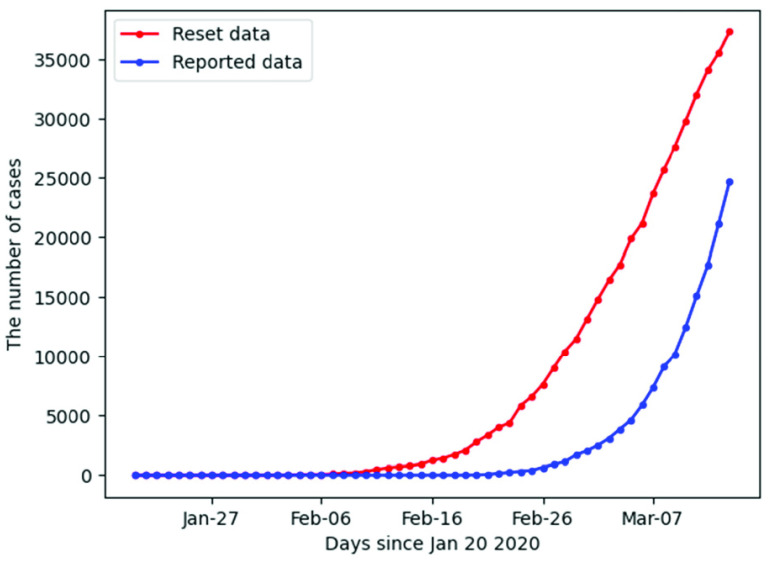
Resetting the initial conditions of the epidemic process: Reported and reset cases calculated by translating the number of confirmed, recovered and death cases.

Transmission of the disease depends on many factors such as the rate of contacts in the host population, the probability of infection being transmitted during contact, the duration of infectiousness, etc. [27] On this basis, using the reported data with the cumulative confirmed, recovered and death cases [25], the cumulative number of the actual infected cases and death rate from reset data would be higher than reported. The number of confirmed cases based on reset data is shown in Fig. 1 in comparison to the observed (that is, reported) cases. Thus, by such resetting the initial conditions of the epidemics, we can further predict the infection curve of the epidemics according to the SIR model, that is from $\beta (t)$, and $\gamma (t)$, calculated from the data. Specifically, once we have the reset data, the methodology used by Zhong *et al.* [15] for parameter estimation can be further applied.

### C. Model of Epidemic Spread

To estimate the early dynamics of transmission of the SARS-CoV-2 virus in Italy, we have used a mathematical dynamic model, so called SIR (*Susceptible Infected Removed*). This modelling process for spread of disease divides the (fixed) population of $N$ individuals into three groups which may vary as a function of time, $t$ [28]:

• S(t), the number of susceptible individuals;
• I(t), the number of infected individuals;
• R(t), the number of recovered (and dead) individuals.

The SIR model describes the change in the population of each of these groups in terms of two parameters, $\beta $ and $\gamma $. $\beta $ describes the effective contact rate of the disease; an infected individual comes into contact with $\beta N$ other individuals per unit time (of which the fraction that are susceptible to contracting the disease is S/N). $\gamma $ is the mean recovery rate in a population: that is, $1/\gamma $ is the mean period of time during which an infected individual can pass it on [29].

The differential equations describing this model were first derived by Kermack and McKendrick [30]:

\begin{align*} {dS} \mathord {\left /{ {\vphantom {dS {dt}}} }\right. } {dt}=&{-\beta S(t)I(t)} \mathord {\left /{ {\vphantom {=-\beta S(t)I(t) N}} }\right. } N \tag{1}\\ {dI} \mathord {\left /{ {\vphantom {dI {dt}}} }\right. } {dt}=&{\beta S(t)I(t)} / N-\gamma I(t) \tag{2}\\ {dR} \mathord {\left /{ {\vphantom {dR {dt}}} }\right. } {dt}=&\gamma I(t)\tag{3}\end{align*}

While in this model $\beta $ and $\gamma $ are constant values, treating the infection and recovery rates as a time-varying variable can capture the dynamical process of the epidemic transmission including the natural processes and the human intervention [13], which most of the European countries have experienced during the March and April 2020.

According to [15], the above equations (1)–(3) can be discretized as:

\begin{equation*} I\left ({t+\Delta t }\right)=I\left ({t }\right)+\left ({\beta -\gamma }\right)(t)I(t)\Delta t\tag{4}\end{equation*}

The following expressions are defined for empirically calculating the infection rate $\beta \left ({t }\right)$ as well as the for the recovery rate $\gamma \left ({t }\right)$;

\begin{align*} \beta \left ({t }\right)=&({I\left ({t }\right)-I(t-\Delta t)} \mathord {\left /{ {\vphantom {I\left ({t }\right)-I(t-\Delta t) {I(t-\Delta t)}}} }\right. } {I(t-\Delta t)} \tag{5}\\ \gamma \left ({t }\right)=&({R\left ({t }\right)-R(t-\Delta t))} \mathord {\left /{ {\vphantom {R\left ({t }\right)-R(t-\Delta t)) {I(t-\Delta t)}}} }\right. } {I(t-\Delta t)}\tag{6}\end{align*}

where $I(t)$ is number of infected cases on day $t$, $I(t-\Delta t)$ is number of infected cases on day $t-\Delta t$, $R(t)$ is a number of closed cases (that is, a sum of both recovered and death cases) on day $t$, and $R(t-\Delta t)$ is a number of closed cases on day $t-\Delta t$.

These values were determined by the reported data directly, and as such, they allow for event monitoring as the epidemic develops and detecting the efficacy of any intervention measure dynamically.

## III. Empirical Results

For most host-pathogen models of the infectious disease, the key parameters are difficult to estimate because natural processes are stochastic and transmission events are influenced by other parameters than what can be included in a model of epidemic spread [31]. Thus, large datasets are often needed to reach a good estimate. Also, it is not possible to study the precision of the estimated infection and removal rates on data from a real population because this implies that the real rates are known [13].

In this section, the empirical results from the set of experiments based on the reported data [2], [25] and the implemented algorithm COVID-19-SHIFT (Table 1) are presented. The epidemiological data of the COVID-19 contains a set of variables and their values for both reported and reset data; the cumulative number of the infected individuals (I/I’), the cumulative removed infectives (R/R’– these include the death and recovered cases), the infection rate ($\beta /\beta $’) and the removal rate ($\gamma /\gamma $’). Table 2 shows an excerpt from these epidemiological data for the relevant initial time period. It can be noted that at a particular point in time the reset and reported number of cases meet.

**TABLE 2:** An Excerpt From the Dataset of Cumulative Infection Cases (Cum. I(t)), Removed Cases (Cum. R(t)), Infection Rate ($\beta\left({t }\right)$) and Removal Rate ($\gamma\left({t }\right)$) From the Reported Data. From These Values, Using the COVID-19-SHIFT Algorithm, Cumulative Infection Cases (Cum. I’(t)), Infection Rate ($\beta'\left({t }\right)$) and Removal Rate ($\gamma'\left({t }\right)$) are Calculated

Date	Cum. I(t)	Cum I’(t)	R(t)	$\beta \left ({\mathrm {t} }\right)$	$\gamma \left ({\mathrm {t} }\right)$	$\beta '\left ({\mathrm {t} }\right)$	$\gamma '\left ({\mathrm {t} }\right)$
2020–02–06	2	59	0	0	0	0.072727	0
2020–02–07	3	65	0	0.5	0	0.101695	0
2020–02–08	3	103	0	0	0	0.584615	0
2020–02–09	3	173	0	0	0	0.679612	0
2020–02–10	3	192	0	0	0	0.109827	0
2020–02–11	3	313	0	0	0	0.630208	0
2020–02–12	3	468	0	0	0	0.495208	0
2020–02–13	3	604	0	0	0	0.290598	0
2020–02–14	3	698	0	0	0	0.155629	0
2020–02–15	3	769	0	0	0	0.101719	0
2020–02–16	3	921	0	0	0	0.197659	0
2020–02–17	3	1238	0	0	0	0.344191	0
2020–02–18	3	1409	0	0	0	0.138126	0
2020–02–19	3	1720	0	0	0	0.220724	0
2020–02–20	3	2065	1	0	0.333333	0.200581	0.000581
2020–02–21	20	2791	2	8.5	0.5	0.351744	0.000484
2020–02–22	62	3336	3	2.333333	0.055556	0.195411	0.000359
2020–02–23	155	3991	4	1.576271	0.016949	0.19652	0.0003
2020–02–24	229	4357	9	0.490066	0.033113	0.091798	0.001254
2020–02–25	322	5796	12	0.422727	0.013636	0.330957	0.00069
2020–02–26	400	6552	16	0.251613	0.012903	0.130705	0.000692
2020–02–27	650	7599	63	0.651042	0.122396	0.16019	0.007191
2020–02–28	888	8920	68	0.405451	0.008518	0.175292	0.000663
2020–02–29	1128	10335	80	0.292683	0.014634	0.159851	0.001356
2020–03–01	1694	11299	118	0.540076	0.03626	0.094003	0.003706
2020–03–02	2036	12958	202	0.217005	0.053299	0.148377	0.007513
2020–03–03	2502	14652	240	0.254089	0.02072	0.1328	0.002979
2020–03–04	3089	16191	384	0.259505	0.06366	0.106786	0.009992
2020–03–05	3858	17597	563	0.284288	0.066174	0.088948	0.011324
2020–03–06	4636	19522	721	0.236115	0.047951	0.113009	0.009276
2020–03–07	5883	20847	823	0.318519	0.026054	0.070475	0.005425
2020–03–08	7375	23370	989	0.294862	0.032806	0.125999	0.00829
2020–03–09	9172	25279	1188	0.281397	0.031162	0.085296	0.008891
2020–03–10	10149	27198	1636	0.12237	0.056112	0.079656	0.018596
2020–03–11	12462	29212	1873	0.271702	0.02784	0.078789	0.009272
2020–03–12	15113	31330	2275	0.250354	0.037964	0.077472	0.014704
2020–03–13	17660	33492	2706	0.198395	0.033572	0.074411	0.014834
2020–03–14	21157	35016	3408	0.23385	0.046944	0.049503	0.022803
2020–03–15	24747	36640	4145	0.202265	0.041523	0.051379	0.023317
2020–03–16	27980	38966	4908	0.156927	0.037035	0.07158	0.023481
2020–03–17	31506	41655	5445	0.152826	0.023275	0.078954	0.015767
2020–03–18	35713	43997	7004	0.161429	0.059821	0.064678	0.043054
2020–03–19	41035	46598	7846	0.185377	0.029329	0.070311	0.022761
2020–03–20	47021	49175	9162	0.180361	0.039652	0.0665	0.03396

Because the reset number of confirmed cases is much higher than the reported, from Table 2 it can be observed that the respective values of $\beta $ and $\gamma $ are much higher than $\beta $’ and $\gamma $’. The mean value of $\beta $ is 61% higher than the mean of $\gamma $’, and the mean value $\beta $ is 83% higher than the mean of $\gamma $’ during the period until March 20, 2020 (after which the reported number is reached).

The course of the epidemic could change dynamically subject to various medical and administrative interventions [13]. Therefore, one of the goals of this study is to elaborate on whether the government intervention worked as expected, i.e. if the spread of infection was reduced by the measures. For that reason, we have split our data into two subsets of interest. The first one ranges from Feb 6, 2020, and the second subset ranges from March 9, 2020 when the government intervention was introduced in Italy. Thus, two subsets can be understood as the low- and high-level anti-epidemic measures against the SARS-CoV-2 virus transmission in model simulation, respectively.

From the basic assumptions of epidemic transmission [32], the parameters $\beta $ and $\gamma $ satisfy a monotonic decreasing and increasing function, respectively. In order to predict the future number of infectives, the infection rate is often specified as a constant or a time-varying analytical function [33]. According to these assumptions, a study based on COVID-19 in the mainland China [15], has confirmed that the exponential model is an appropriate fit for the infection rate, and a constant value is an appropriate fit for the removal rate.

**FIGURE 2.**
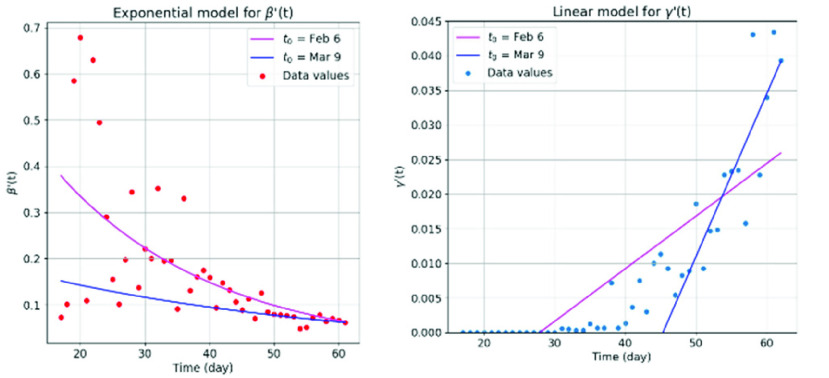
Fitting the (a) exponential ${\beta '(t)}$ and (b) linear ${\gamma '}\left ({{t} }\right)$ from the reset data using subsets $\text{t}_{0}=$ Feb 6 and $\text{t}_{0}=$ Mar 9.

However, to adopt one constant number to describe the removal rate of an epidemic development is not straight forward, for the dynamic of the epidemic may exhibit different transmission pattern in different phases of the event [34]. Therefore, in our first experiment, we fitted a time-varying function to both $\beta '(t)$ and $\gamma '(t)$. More specifically, we fitted an exponential model to $\beta '(t)$ as shown in Fig. 1a, and a linear model to $\gamma '(t)$ as shown in Fig 1b. From Fig. 2a, it can be noticed that the fitted exponential functions show a large difference between two subsets of data.

The exponential function for the $t_{0} =$ *Feb 6* subset (low-level anti-epidemic measures) is much steeper than the $t_{0} \,\,=$ *Mar 9* subset (high-level anti-epidemic measures), which may result in unrealistically strong prohibition of the infective number [15]. From Fig. 2b, it can be observed that the linear function for the $t_{0} =$ *Feb 6* subset is less steep than the $t_{0} \,\,=$ *Mar 9* subset, because in the first subset there were a few days without removed cases. These models provide a basis for predicting new cases of infection.

**FIGURE 3.**
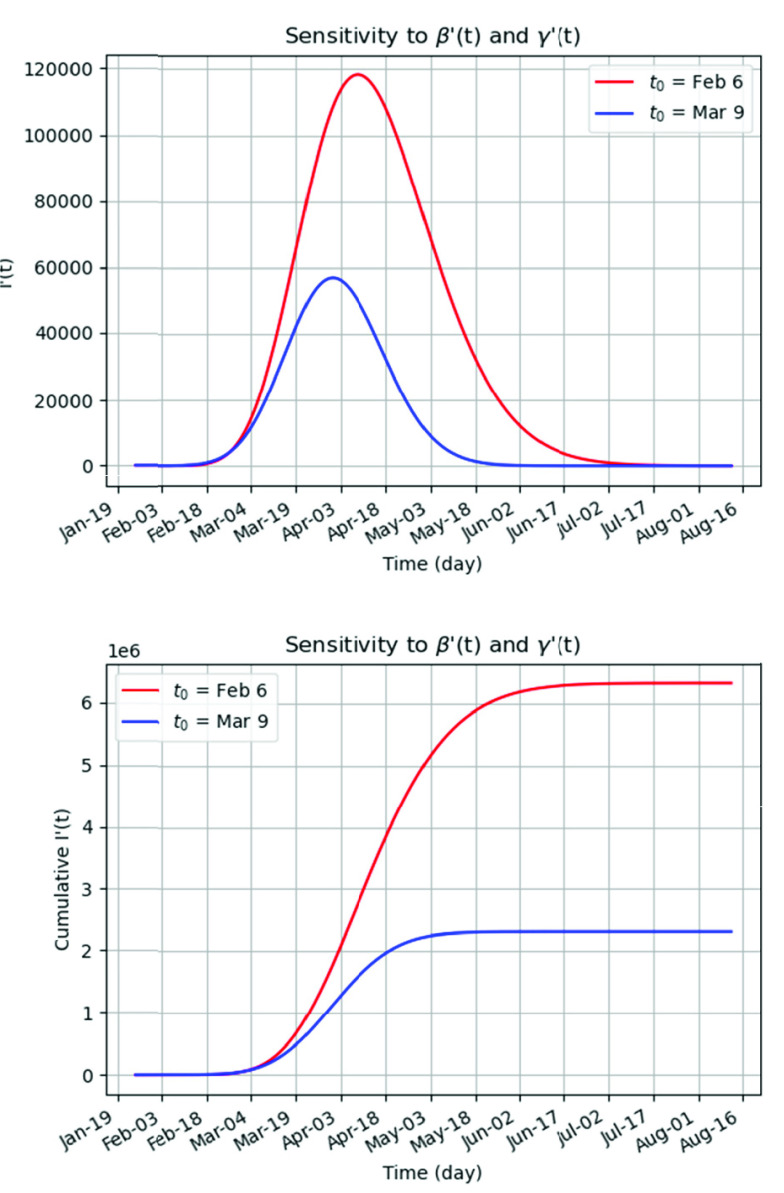
The prediction of the infected cases of the COVID-19 using the subsets $\text{t}_{0}=$ Feb 6 and $\text{t}_{0}=$ Mar 9: (a) the sensitivity of the number of the infectives I’(t) to the fitted functions of $\beta $’(t) and $\gamma $’(t) shown in Fig 2. (b) the sensitivity of the number of cumulative infectives I’(t) to the fitted functions of $\beta $’(t) $\gamma $’(t) shown in Fig 2.

Drawing on the calculations presented in Table 2, the fitted models for $\beta '(t)$ and $\gamma '(t)$, as well as the equation (4), a time-based number of new infected cases is shown in Fig. 3 to describe the complete process of epidemics. Compared to the low-level anti-epidemic spread scenario ($\text{t}_{0}=$ Feb 6), the high-level anti-epidemic measures ($\text{t}_{0}=$ Mar 9) reduce the peak of the infected cases by 52%, and the cumulative cases by 63%.

The high-level anti-epidemic spread scenario has the inflection point on March 28 with the peak of 56,962 infectives and 2,313,896 cumulative infectives. The low-level anti-epidemic curve has the inflection point on April 8 with the peak of 118,428 infectives and 6,327,057 cumulative infectives. The fading out period for the high-level strengthened measures curve (with number of infectives less than 1,000) starts on May 10, while for the low-level anti-epidemic spread it starts on June 30. The high-level anti-epidemic measures seem to have a strong effect on both the fading-out date and the number of the infected cases.

**FIGURE 4.**
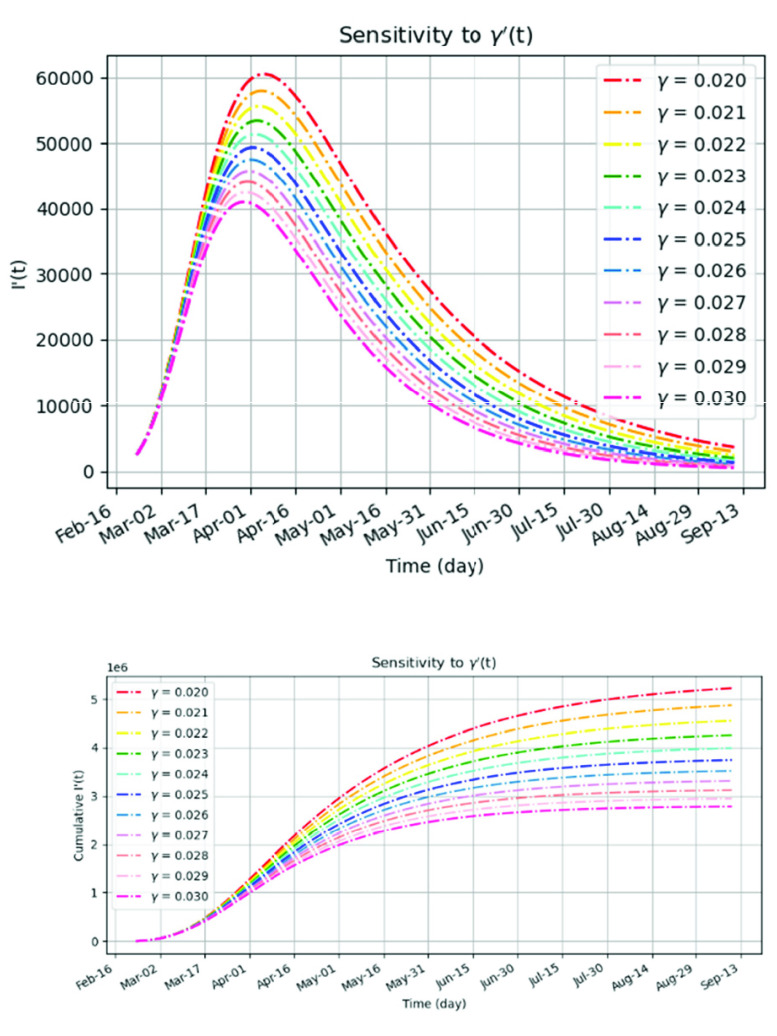
The prediction of the infected cases of the COVID-19 using the subset $\text{t}_{0}=$ Mar 9: (a) the sensitivity of the number of the infectives I’(t) to the exponential $\beta $’(t) and constant $\gamma $’(t). (b) the sensitivity of the number of cumulative infectives I’(t) to the exponential $\beta $’(t) and constant $\gamma $’(t).

The second experiment was conducted using the constant values for the removal rate, while the same exponential model for the infection rate remained. It can be observed that the I’(t) from Fig. 4a has wider ‘bell-shape’ and is not as symmetrical as I’(t) from Fig. 3a, which implies a longer fading out period. The reason is that the linear model of the $\gamma $(t) is more tightened, yet appropriate for modelling strong application of administrative measures, while the constant $\gamma $ is more suited to the natural process of the epidemics. It can be noted that I’(t) in Fig. 4 is highly sensitive to the changes of $\gamma $ of a size of a magnitude of 0.001.

The change of 3% of $\gamma $ ($\Delta \gamma =0.001$) implies the change in the number of infectives by at least 8%. Thus, the increase of $\gamma $ is effective in prohibiting the infected number [15].

It can be noted that a larger removal rate or stronger administrative measures significantly shorten the duration of the epidemic outbreak, but overall, the forecast scenarios’ peak and recovery phases are based on the assumptions that the government continues to enforce strict social-distancing and other required measures. If these measures are lifted too early, there could be very easily seen a second wave of exponential increase in cases.

In these experiments, the estimate of the number of infectives ranges from more than 2 ($\gamma =0.03$) up to 5 ($\gamma =0.02$) million people, which is significantly higher than those suggested by the official reports. The possible explanation is that the country may not be catching many of the mild cases of COVID-19.

**FIGURE 5.**
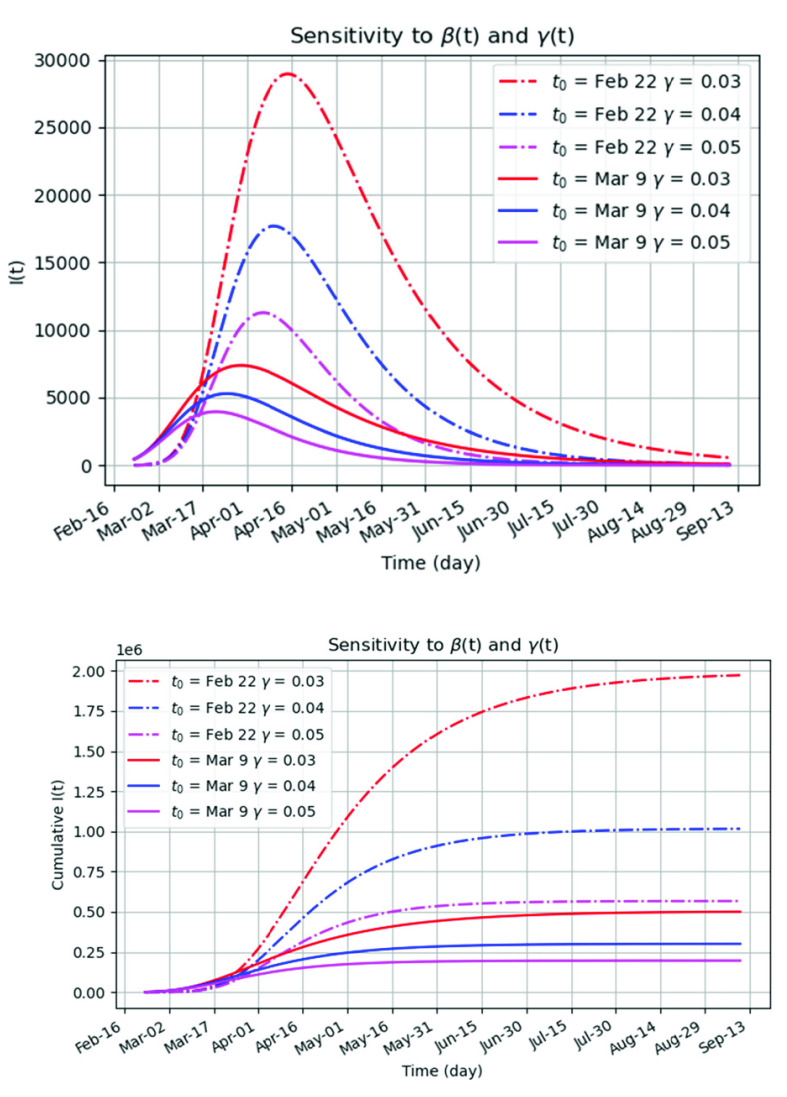
The prediction of the infected cases of the COVID-19 using only reported data: (a) the sensitivity of the number of the infectives I(t) to the exponential $\beta $(t) and constant $\gamma $(t). (b) the sensitivity of the number of cumulative infectives I(t) to the exponential $\beta $(t) and constant $\gamma $(t).

The third experiment we have conducted is based only on reported data to compare it to our approach as shown in the first two experiments. According to Table 2, we fitted $\beta (t)$ to the exponential model, and $\gamma (t)$ to a constant value. Fig. 5 shows the COVID-19 propagation under the low-level and high-level anti-epidemic measures with three different values of $\gamma $ (0.03, 0.04 and 0.05), which may be attributed to low, moderate, and high medical levels of disease treatment. Here, the changes of $\gamma $ are one size of the magnitude higher than those in the previous experiment, so the changes in I(t) are much higher. From Fig 5a, the peak of the infected cases may reach 3,947 - 5,833 (that is, cumulative 196,235 - 501,026) under high-level prevention measures, but 11,281 - 28,948 (that is, cumulative 567,426 - 1,973,006) under the low-level measures.

Compared to low-level anti-epidemic scenarios ($\text{t}_{0}=$ Feb 6), the strengthened measures ($\text{t}_{0}=$ Mar 9) reduce the peak infected cases by 73%, 70% and 64%, and the cumulative cases by 74%, 71% and 65% under low, moderate and high medical levels ($\gamma $), respectively. Thus, it can be concluded that also in this experiment the increase of $\gamma $ is effective in prohibiting the infected number. The fading out period for the high-level anti-epidemic scenario begins by the end of May, while for the low-level anti-epidemic scenario is postponed until July, or even later ($\gamma =0.03$). This confirms that the epidemic is highly sensitive to the medical-service level as the proper medical care can significantly prohibit the propagation of the disease.

It is interesting to notice that the worst scenario in Fig. 5 is closest to the high-level anti-epidemic scenario in Fig. 3. It should be noted that the accuracy of the scenarios is dependent on the accuracy of historical data, which for a newly emerged disease such as COVID-19 has serious data limitations. Limitations include but are not limited to accuracy of testing, testing availability, testing speed, speed of reporting, and accuracy of reporting. In any case, quarantine adherence has an important and notable impact on reducing the outbreak, but some transmission will still occur within households [36].

## IV. Discussion

It is important to note that it might still be early to predict overall outcome of the pandemic due to the complexity and variety of the biomedical and social factors involved [37], however mathematical models can be used as intelligent input during the decision-making process. These models can help predict the health infrastructure and material needs that patients will need in these countries in near future [38].

### A. Generalization of the Proposed Method

Although we have shown a case study of Italy, our proposed method of resetting the initial conditions of the process of epidemic spread may serve as a generalized approach to analyzing epidemiological data from any micro- or macro-location affected by the SARS-CoV-2 virus. The steps of the COVID-19-SHIFT algorithm (Table 1.) are independent of a region or country since the virus affects humans at almost any geographical location, i.e. the virus has spread worldwide [2].

Moreover, the proposed method might be applicable to similar epidemiological phenomena if the course of the disease is known, such that these parameters can be injected into the COVID-19-SHIFT algorithm.

In addition, it is possible to introduce the reset initial conditions of an epidemic process into other mathematical models besides the SIR model. In other words, the proposed model can be extended from various aspects. One such extension is to separate certain subgroups from the groups of susceptible, infected, or recovered cases, and then to conduct additional analysis.

### B. Efficiency of the Proposed Method

It should be noted that the efficiency of our approach is highly dependent on the accuracy of the available data, which is a challenging task itself due to different SARS-CoV-2 testing policies in different countries, e.g. the number of daily tests, type of the tests, etc.

One of the efficiency metrics might be a comparison of the theoretical results from a predictive model to the results of the actual serological tests for detection of specific antibodies against the SARS-CoV-2 virus. Serological tests may provide information on who has COVID-19, who has been infected, and who may have an immunity [39]. Serological surveys are a valuable tool to assess the extent of the epidemic, given the existence of asymptomatic cases and little access to diagnostic tests [40]. Although currently there are only a few available studies representing the general population (Spain, Switzerland, China and USA), they confirm that the number of infected cases was higher than officially reported [41]–[44].

The first Italian survey on healthcare staff members suggested a high prevalence of immune individuals (i.e. at least among at-risk categories), as the results demonstrated the presence of antibodies against SARS-CoV-2 in 17.2% of subjects [39].

In Spain, the first COVID-19 case was reported on Jan 31. The first death occurred on Feb 13, but it was not diagnosed post-mortem until early March [2]. It is interesting to observe that the Mediterranean countries Italy and Spain have many similarities such as highly social citizens, fair weather, densely populated cities, physically affectionate social interactions, and large elderly populations [38]. Thus, it is expected that Italian nationwide serological survey would possibly provide similar results such as those from Spain. A recent study conducted in Spain [41], showed a nationwide seroprevalence of 5%.

In addition, a study conducted in Switzerland [42], showed a seroprevalence of 10.8% based on data from Geneva. A study done in Wuhan, China [43], reported a seroprevalence of 3.8%. It is also indicative that Sweden, as a country without strict lockdown measures, reported a prevalence of 7.3% [45]. In the community seroprevalence study in Los Angeles County, the prevalence of antibodies to SARS-CoV-2 was 4.6%, which is substantially greater than the cumulative number of confirmed infections [44].

On the other hand, the efficiency of our approach, or how well our model performs, is further elaborated using comparison with other models from the same pandemic context. The following studies demonstrate findings that are in-line with our findings, either in terms of predicting higher number of infected cases than reported, or in terms of the beginning of the epidemic spread.

A study based on the SIR model and data in Wuhan, China [47], suggested that “the actual number of infected cases could be greater than the number of reported cases”.

The SEIR (*Susceptible Exposed Infected Recovered*) model [48], has also confirmed that “the actual number of cases were likely much higher than reported”, as well as the earlier occurrence of the COVID-19 disease than reported in mainland China, i.e. “the predicted start of the epidemics happened in November 2019, with hundreds of infected cases already present in early December”.

A study that also fitted a SEIR model of infection [49], has estimated that only 5.1% of infected cases in Wuhan were reported. It has been also estimated by another study [50] that the ascertainment rate of infection in Wuhan was 9.2% informing us that 90% of the cases were potentially undiagnosed or unreported.

In a study based on the $\theta $-SEIHRD model [51], it was found that the final number of undetected cases was estimated by represented around 52% of the total number of cases. It is concluded that, despite the relative control of the epidemic in China, there might still exist an undetected source of infected people that could have caused an increase of the epidemic in a near future, if the control measures were significantly relaxed. The $\theta $-SEIHRD model is based on the Be-CoDiS model [52] and takes into account the different sanitary and infectiousness conditions of hospitalized people.

To summarize, due to the high proportion of asymptomatic or mild COVID-19 infections (approximately 80%) [53], data restricted to laboratory-confirmed cases do not capture the true extent of the spread or burden of the virus [40]. Thus, it remains a challenging task to both predict the spread of the infection as well as to validate efficiency of the model.

### C. Limitations of the Study

The predictive ability of the model is limited by the accuracy of the available data and by the level of abstraction used for modeling the epidemic spread.

The proposed model has not taken into account the impact of factors such as population birth rate and natural mortality. Also, it has assumed that people make connections and communicate with each other at random, which is not the case in a real life [54]. Other demographical factors, such as gender or age are also not considered in our study. For example, a recent study [55] proposed the impact of age and gender on the death count using mathematical modelling and showed that this virus is largely affecting the elderly.

Other external factors, such as spatial, environmental, and/or meteorological were not considered by the model but pose a challenging task for future avenues of the research.

Additionally, the current model is only suitable for countries or territories with a relevant number of people infected by COVID-19, where local transmission is the major cause of the disease spread. The between-country spread has not been modeled in this work.

The traditional SIR model is a high-level abstraction model and cannot fully describe the impact of government measures on different populations. According to the actual situation of the epidemic, the population can be divided into additional different categories to comply with the current spread of COVID-19, such as *asymptomatic*, *suspected*, *quarantined*, *hospitalized* [56]. In addition, the outbreak of the disease does not cause lifetime immunity, which can result in a constant rate of illness throughout time, after its transient period. Also, some unforeseeable factors may affect these estimated data in our study, such as super-spreaders exist.

Overall, the proposed approach is going to work if SARS-CoV-2 virus behaves in an expected way, within our current grasp of understanding [57]. However, if the virus mutation occurs causing re-infection, or if new waves of infection introduce large numbers of new cases [58], the context might have changed.

## V. Conclusion

In today’s global society, the example of COVID-19 outbreak has shown how quick the infectious disease can spread across the world, fueled by the rapidity with which we travel across borders and continents [35]. Even if there is a certain kind of agreement among most of the governments on application of some level of anti-epidemic measures, there is still tremendous uncertainty on what exactly needs to be done to stop the SARS-CoV-2 virus [36]. Currently available data [2] make it possible to analyze passed events as well as to predict the near future based on the trends and the acquired knowledge from the past.

Our study has joined the research efforts on estimating the real number of the infective cases in a population, using a case study from Italy. Possible reasons for such rapid growth of infections in Italy include more timely caution and preventative measures were not taken [38]. Also, the number of infections during Jan 31 - Feb 20 could have been under-reported due to underdiagnosis, given subclinical or asymptomatic cases [39]. Data coming from various sources [2], [8], [25] suggested that Italy has started to flatten the infection curve at the beginning of April, and the social activities have been gradually restarted during May [39].

In our approach to modelling epidemic spread, the infection and recovery rates are data driven, so the accurate data is the basis for precise estimation of these rates. Therefore, the increased size of the data or samples will decrease the uncertainty for the estimated rates. In addition, our predictions are strongly determined by the applied anti-epidemic measures and the medical treatment against the COVID-19.

To decrease the risk of a second wave in places where the first wave is controlled by robust social distancing, governments would need to consider mass testing, contact tracing, and quarantines for those infected until a vaccination is available, mass produced, and distributed widely [35].
